# Supplementary figures and images for: Fungemia in Hospitalized Adult Patients with Hematological Malignancies: Epidemiology and Risk Factors
Source: J Fungi (Basel). 2023 Mar 24;9(4):400. doi: 10.3390/jof9040400 (PMC10142635; doi:10.3390/jof9040400)

Supplementary Figure 1 flow-chart of patient selection.

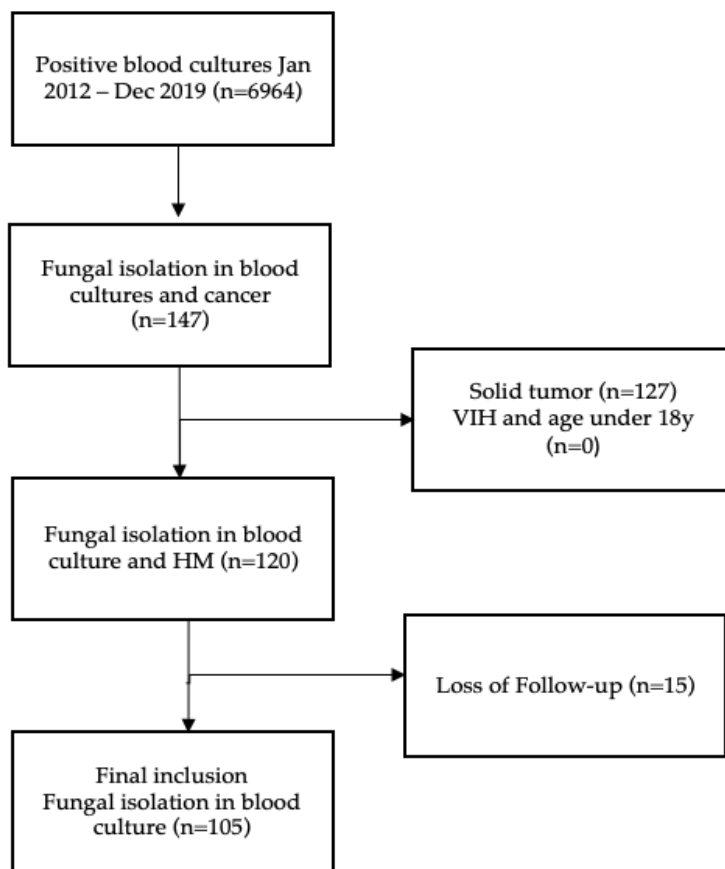

Supplement: Supplementary file 1 [file jof-09-00400-s001.zip › jof-2213473-supplementary.pdf]
